# Origins, Development, Current Challenges and Future Directions with Activated Prothrombin Complex Concentrate for the Treatment of Patients with Congenital Haemophilia with Inhibitors

**DOI:** 10.1055/a-1159-4273

**Published:** 2020-07-27

**Authors:** Hans H. Brackmann, Wolfgang Schramm, Johannes Oldenburg, Viridiana Cano, Peter L. Turecek, Claude Négrier

**Affiliations:** 1 Haemophilia Center, Institute of Experimental Haematology and Blood Transfusion, University of Bonn, Bonn, Germany; 2 Rudolf Marx-Stiftung für Hämostaseologie, Universität München and Bluterbetreuung Bayern e. V. (BBB) - Germany; 3 Shire International GmbH, a Takeda company, Zürich, Switzerland; 4 Baxalta Innovations GmbH, a Takeda company, Vienna, Austria; 5 Haemophilia and Thrombosis Centre, Louis Pradel Hospital, University Claude Bernard Lyon 1, Lyon, France

**Keywords:** congenital haemophilia, inhibitors, bypassing agents, FEIBA, aPCC

## Abstract

Congenital haemophilia A (HA) is caused by deficiency of coagulation factor VIII (FVIII) activity, leading to spontaneous or traumatic bleeding events. While FVIII replacement therapy can treat and prevent bleeds, approximately 30% of patients with severe HA develop inhibitor antibodies that render FVIII replacement therapy ineffective. The bypassing agents (BPAs), activated prothrombin complex concentrate (aPCC) and recombinant activated FVII, first approved in 1977 and 1996, respectively, act to generate thrombin independent of pathways that involve factors IX and VIII. Both may be used in patients with congenital haemophilia and inhibitors (PwHIs) for the treatment and prevention of acute bleeds and quickly became standard of care. However, individual patients respond differently to different agents. While both agents are approved for on-demand treatment and perioperative management for patients with congenital haemophilia with inhibitors, aPCC is currently the only BPA approved worldwide for prophylaxis in PwHI. Non-factor therapies (NFTs) have a mechanism of action distinct from BPAs and have reported higher efficacy rates as prophylactic regimens. Nonetheless, treatment challenges remain with NFTs, particularly regarding the potential for synergistic action on thrombin generation with concomitant use of other haemostatic agents, such as BPAs, for the treatment of breakthrough bleeds and in perioperative management. Concomitant use of NFTs with other haemostatic agents could increase the risk of adverse events such as thromboembolic events or thrombotic microangiopathy. This review focuses on the origins, development and on-going role of aPCC in the evolving treatment landscape in the management of PwHI.

## Introduction

Congenital haemophilia A (HA) and B (HB) are bleeding disorders characterised by a deficiency of blood clotting factor VIII (FVIII) or factor IX (FIX), respectively. 1 The type of FVIII/IX mutation present is a major determinant of severity and bleeding tendency. 1 Severe cases present with bleeding and joint bleeds from early childhood, which, without appropriate treatment and prevention, can result in irreversible joint damage and chronic arthropathy. 2

Strides have been made in the management of congenital haemophilia over recent decades, including the introduction of plasma-derived and recombinant clotting factor products, use of prophylaxis as standard of care for bleeding prevention, and appropriate surgical management. 3 4 5 6 7 Such therapy has led to improvements in the health of patients with haemophilia by suppressing the onset of joint damage and arthropathy, preventing life-threatening bleeds, and improving patient quality of life. 8 9 Nevertheless, treatment challenges remain. First, for patients receiving FVIII/IX products, intravenous infusion is required up to every 2 days for patients with severe HA and at least twice weekly for those with severe HB. 10 11 Although high infusion frequency can be reduced with the use of extended half-life products, the frequency can still be burdensome. 12 Second, treatment can be complicated by the development of alloantibodies (inhibitors) that bind to FVIII or FIX, preventing its haemostatic action. 13 Such antibodies can neutralise therapeutically administered factor replacement products, and occur in up to 25 to 40% of severe HA patients, 5 to 15% of moderate/mild HA patients and 1 to 5% of patients with severe HB. 14 Anaphylactic reactions and nephrotic syndrome are also not uncommon in patients with HB and inhibitors. 15 16

The aetiology of inhibitor development is multifactorial, including both genetic and treatment-related risk factors. 17 18 19 20 Presence of inhibitors is associated with reduced treatment efficacy, increased occurrence of life-threatening bleeds and severe joint damage, which can lead to poor quality of life for patients, family and caregivers; higher morbidity and mortality rates; and increased healthcare costs. 21 22 23 Recommended treatment of patients with congenital haemophilia and inhibitors (PwHIs) has focused on eradicating inhibitors using immune tolerance induction (ITI) therapy. 3 4 5 6 7 24 25 ITI regimens vary and can be used with or without bypassing agents (BPAs) for the treatment of breakthrough bleeding, surgical setting and prophylaxis. 7

BPAs were developed to ‘bypass’ the factors blocked by inhibitors, and function by generating thrombin via pathways that do not require activation of FVIII or FIX. 26 Two BPAs are currently available: activated prothrombin complex concentrate (aPCC, FEIBA [factor eight inhibitor bypass activity]; Takeda, Lexington, Massachusetts, United States) and recombinant activated FVII (rFVIIa, NovoSeven; NovoNordisk, Bagsvaerd, Denmark). Both compounds have been approved for on-demand treatment and perioperative management for PwHIs, while aPCC is the only compound approved worldwide for prophylaxis in PwHI. 27 28 29

Both aPCC and rFVIIa have efficacy rates >80% in the control of acute bleeding events, with comparable tolerability and low rate of thrombotic complications, as concluded by a Cochrane systematic review. 30 The choice of BPA for on-demand treatment may be driven by several factors, including burden of the infusion due to volume and infusion time, experience of treater and/or patient preference. 26 Furthermore, individuals may show a better response to one agent over another, as reflected in the FEIBA NovoSeven Comparative (FENOC) study, 31 in which 32% of patients reported efficacy for either aPCC or rFVIIa at 6 hours post-treatment. 31 Achievement of good haemostatic efficacy within the first few hours of a bleed can reduce the risk of cartilage destruction; therefore, selection of the most appropriate BPA for each individual is important. 31

Treatments with different mechanisms of action that aim to address the challenges of treating PwHI are in development. Here, we review the role of aPCC in an evolving treatment landscape for patients with congenital HA with inhibitors.

## Development of Activated Prothrombin Complex Concentrate

The clinical use of prothrombin complex concentrates (PCCs) was extended early beyond their basic use as a substitution therapy for patients with prothrombin complex protein deficiencies, particularly for those with a FIX deficiency (HB), to the treatment of those with inhibitory antibodies against FVIII and FIX. However, the clinical use of PCCs remained a niche indication for many years. Only six treatment episodes were reported until 1977. 32 33 34 35 For a long time, the clinical success of PCCs in the management of patients with inhibitors was attributed to the activated prothrombin complex enzyme in addition to the zymogen content of the concentrates. 36 Therefore, in the early 1970s, the so-called auto-FIX concentrates were developed as a new therapeutic approach for treating HA patients with inhibitors. 37 The independent development of commercial aPCCs began around 1970 by two laboratories in parallel: Hyland Laboratories in the United States developing anti-inhibitor coagulant complex (Autoplex) and Immuno AG in Austria developing aPCC (FEIBA), the latter of which became the mainstay of treatment for patients with inhibitors.

aPCC is a plasma-derived, vapour-heated and nano-filtered (35 nM) concentrate of primarily vitamin K-dependent clotting factors (FII, FVII, FIX and FX) in both their zymogen and active forms ( Table 1 ) that is proposed to act at cellular surfaces near the site of injury. 38 It has been commercially available since 1977 to bypass the need for FVIII and FIX and to control and prevent bleeding in PwHI. 39 It is approved in over 80 countries and is indicated for control of spontaneous bleeds, perioperative management and routine prophylaxis for patients with congenital HA or HB with inhibitors and for the treatment of spontaneous bleeds and perioperative management for patients with acquired HA. Please note that indications do vary by country. 27 40

**Table 1:** Haemostatic components of aPCC

	Units per 1 U of FEIBA a
Prothrombin (factor II)	1.3 ± 0.3
Thrombin	0.01 ± 0.004
Factor VII	0.9 ± 0.1
Factor VIIa	1.5 ± 0.2
Factor IX	1.4 ± 0.1
Factor IXa	approx. 0.0006
Factor X	1.1 ± 0.2
Factor Xa	0.06 ± 0.002
Factor VIII	0.03–0.1
Factor V	approx. 0.6
Protein C	1.1 ± 0.2
Protein S	approx. 0.4

Note: Activated prothrombin complex concentrate (aPCC) composition based on data obtained during the release procedure of aPCC and measured in the research laboratories at Baxter as described in the methods. 41 58

a One unit of factor eight inhibitor bypass activity (FEIBA) is defined as the amount of FEIBA capable of shortening the clotting time of high-titre FVIII inhibitor plasma by 50%.

**Fig. 1:**
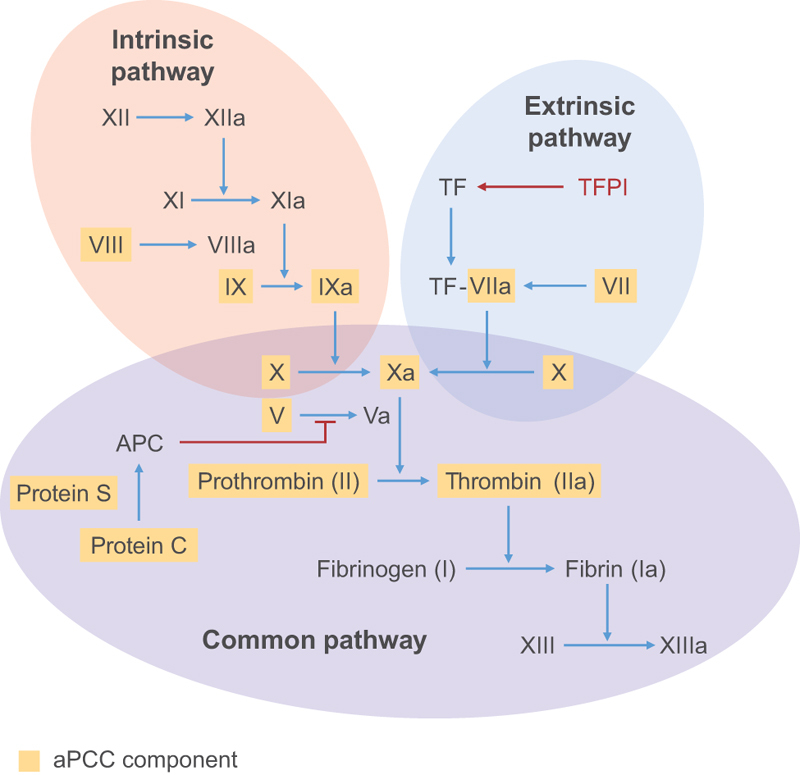
Mechanism of action for activated prothrombin complex concentrate (aPCC). APC, activated protein C; TF, tissue factor; TFPI, tissue factor pathway inhibitor.

The mechanism of action of aPCC is multi-site and involves three main steps ( Fig. 1 ). 41 First, FII–FXa complex triggers immediate thrombin generation (TG) on the membrane surface of tissue-factor-bearing cells and activated platelets, bypassing the haemostatic cascade and prompting initial fibrin clot formation. In addition, thrombin-mediated feedback reactions lead to activation of other coagulant components and to further platelet activation. Second, endogenous anti-tissue factor pathway inhibitors slow down the reactions on tissue-factor-bearing cells. Zymogen and procoagulant enzyme components of aPCC directly and indirectly amplify TG on activated platelet surfaces resulting in a burst of thrombin. Due to the long half-life of the zymogens, circulating substrate levels are elevated, leading to long-lasting haemostasis that prevents the recurrence of bleeds. Third, aPCC contains the natural clotting inhibitors protein C and protein S. 41 A balanced increase in these factors down-regulates coagulation and, thus, might help to prevent thrombotic effects in absence of another procoagulant trigger. 38 42

## aPCC in the Management of PwHI

aPCC has demonstrated control of bleeding episodes in clinical trials and in over 40 years of real-world usage in patients with congenital and acquired haemophilia with FVIII or FIX inhibitors across a range of ages, from toddlers to elderly patients, and in different clinical settings as on demand, 31 43 44 45 46 surgical, 45 47 48 prophylactic 28 44 49 50 51 and concomitant with ITI therapy. 52 53 54 55 56 A post-authorisation safety study of PwHI receiving aPCC as prophylaxis or as on-demand treatment reported the occurrence of 3 treatment-related serious adverse events (AEs) in 3 (3.7%) patients and 6 treatment-related non-serious AEs in 5 patients (6.2%). One deep vein thrombosis was reported in an elderly patient; no other reports of thromboembolic events (TEEs) or thrombotic microangiopathy (TMAs). 28 A meta-analysis of studies in PwHIs reported no TEEs with long-term aPCC prophylaxis or under ITI regimen. 57 In this study, the incidence rate of TEEs for on-demand therapy was 5.09 (95% confidence interval [CI]: 0.01–1,795.6) per 100,000 infusions, and the pooled TEE incidence rate in congenital haemophilia patients was <0.01 per 100,000 infusions. 57 No TMAs have been reported with the use of aPCC as monotherapy to date. 48 58 Key clinical and real-world observational studies with aPCC are summarised in Table 2 .

**Table 2:** Summary of aPCC clinical and real-world studies in PwHI

Reference (first author and year)	Study name	Study design	Inclusion criteria	N	Regimen/dose	Duration of treatment	Outcome
Brackmann 1977 53	–	Case study	Patient with HA with inhibitors	1	ITI: initial 3,000 U FVIII + 2,500 U FIX daily, rising to 12,000 U FVIII daily for 10 d, then gradual reductions over 7 mo to 3,000 U FVIII + 1,000 FIX (aPCC)	7 mo	No demonstrable inhibitor after 7 mo of treatment
Sjamsoedin 1981 120	–	Randomised, double-blind, clinical	Patients with HA with inhibitors	15	On demand: 88 U/kg aPCC after bleed or prothrombin complex concentrate; then post 12 h for muscle bleed or post 6 h for mucocutaneous bleed, if necessary	24 h	aPCC judged as ‘effective’ in 64% episodes; control judged as ‘effective’ in 52% episodes. Bleeding in the same joint and joint mobility significantly improved with aPCC vs. control ( *p* = 0.0085 and *p* = 0.006, respectively)
Hilgartner 1983 43	–	Open-label	Patients with HA or HB and inhibitors aged ≥4 y	49 (46 HA; 3 HB)	On demand: 50–70 U/kg aPCC at 12-h intervals	72 h	93% of 165 bleeding episodes in joints (102 episodes), mucous membranes (20 episodes), muscle and soft tissue (33 episodes), emergency episodes (10 episodes; 3 CNS bleeds and 4 surgical procedures) were controlled. 36% were controlled with 1 infusion in 12 h, 42% by ≥1 infusions in 36 h and 14% in >36 h. No serious side effects
Hilgartner 1990 121	–	Uncontrolled; compared with earlier aPCC study	Patients with HA and inhibitors	41 (106 bleeding episodes)	On demand: vapour-heated aPCC; 50 U/kg for mucus membrane bleeds, 75 U/kg for joint and muscle haemorrhages	Maximum: 36 h	88% episodes were controlled, 79% within 36 h
Brackmann 1996 54	–	Retrospective study	Patients with HA and inhibitors undergoing ITI	81	Prophylaxis under ITI: 100–150 IU/kg FVIII + 50 U/kg aPCC 2 × daily until <1 BU, then 150 IU/kg FVIII 2 × daily	Mean: 10–15 mo	For 22 patients with high responding inhibitors, time to <1 BU was 7 mo, and time to FVIII normalisation was 14.5 mo. For 15 moderate responders, time to <1 BU was 2.4 mo, and time to FVIII normalisation was 10.7 mo
Négrier 1997 63	–	Multicentre retrospective study	Patients with HA or HB with inhibitors receiving aPCC	60	Prophylaxis: 65–510 U/kg/d aPCC, typically as 65–100 U/kg every 6–12 h	NA	Efficacy was judged as ‘good or excellent’ in 81.3% of episodes. Tolerance was assessed as ‘good’ in 98.8% of episodes
Oldenburg 1999 52	–	Open-label	Patients with HA and inhibitors undergoing ITI	60	Prophylaxis under ITI: 100 IU/kg FVIII + 50 U/kg aPCC 2 × daily until <1 BU; 150 IU/kg FVIII 2 × daily until no inhibitor detected and FVIII half-life normalised	Median (range) time to <1 BU: 5.4 (0.2–97.3) mo; to FVIII half-life normalisation: 14.1 (1.8–103.2) mo	Successful immune tolerance was achieved in 52 patients (86.7%); therapy failed in 8 patients (13.3%). The immune tolerance achieved was long-lasting in all 52 patients, with no inhibitor relapse in up to 20 y of follow-up
Ehrlich 2002 122	–	10-y pharmacovigilance (all spontaneously reported thrombotic AEs)	Patients with inhibitors receiving aPCC	–	Safety: equivalent to 3.95 × 10 ^5^ typical infusions distributed worldwide	–	16 AEs documented over 10-y period (incidence of 4.05 per 10 ^5^ infusions). DIC ( *n* = 7) and myocardial infarction ( *n* = 5) were the most frequent. One fatality in an 87-y-old metastatic cancer patient. In 13/16 (81%) patients, known risk factors were present (overdose, obesity, serum lipid abnormalities)
Bui 2002 123	–	Case study	Post-surgical patient supported by ECMO treated with rFVIIa, then switched to aPCC	1	Post-surgery on demand: rFVIIa: 7.8 mg (90 μg/kg) then 4.8 mg. aPCC dose not recorded	NA	Patient died 20 min after aPCC treatment due to aPCC-precipitated thrombosis; clots were noted in the ECMO tubing
Rosenfeld 2002 124	–	Case study	Patients with severe HA and inhibitors receiving prolonged sequential aPCC and rFVIIa	1	On demand: aPCC 75 U/kg daily for 2 d then every 12 h for 1 d; then rFVIIa 90 µg/kg every 2 h for 2 d then every 6 h for 2 d; then aPCC 75 U/kg every 12 h	14 d	Patient developed pulmonary embolism after sequential therapy
Dimichele 2006 48	–	Post-marketing surveillance study	Patients with HA and inhibitors receiving aPCC	63	NA	NA	>4,500 infusions in 204 treatment courses in 63 patients. The incidence of AEs was low (<0.04%). No thrombotic complications were reported. Efficacy was considered good or excellent in 82% of acute treatments and 91% of surgeries
Astermark 2007 31	FENOC	Head-to-head, open-label, crossover equivalency study of aPCC vs.rFVIIa	Patients with HA and inhibitors	48	On demand: 1 dose of aPCC (75–100 U/kg; target dose, 85 U/kg) or 2 doses of rFVIIa (90–120 μg/kg; target dose, 105 μg/kg ×2) IV. Second dose of rFVIIa administered 2 h after the first dose	NA	Efficacy 6 h post-infusion: aPCC and rFVIIa appear to exhibit a similar effect on joint bleeds, although the efficacy between products is rated differently by a substantial proportion of patients. CI for the difference inpercentages of efficacy reported for each product only slightly exceeded the 15% boundary (−11.4 to −15.7%), *p* = 0.059
Schneiderman 2007 125	–	Retrospective chart review	Hospitalised children aged 18 mo to 16 y with severe refractory haemophilia and inhibitors	4 (35 hospital admissions)	On demand: sequential (≤6 h intervals) aPCC (32–80 U/kg) and rFVIIa (103–209 µg/kg)	–	No clinical signs of thrombosis. Resolution of bleeds after a median of 3 d sequential therapy
Ettingshausen 2010 64	–	Long-term prospective study	Paediatric patients (5.4–15 y) with HA and high-responding inhibitors who had failed, interrupted, or refused immune tolerance therapy	7	Prophylaxis: aPCC 50–100 U/kg, at frequencies from 3 times weekly up to twice daily	0.1–1.9 y at start of any therapy; 1.5–11.8 y at start of aPCC prophylaxis	Mean annual spontaneous joint bleed incidence rate: 1.5 (95% CI: 0.7–3.0); no or mild osteoarthropathic alterations; no thrombotic complications, DIC or viral transmission
Leissinger 2011 44	PRO-FEIBA	Prospective, randomised, crossover study	Patients with HA >2 y of age, with high-titre inhibitors	26	Prophylaxis: 85 U/kg ± 15% aPCC on 3 non-consecutive d/wk On demand: 85 U/kg ± 15% aPCC	6 mo	Mean 5.0 bleeding events on prophylaxis vs. 13.1 with on-demand treatment; representing a 62% reduction with prophylaxis vs. on-demand ( *p* < 0.001). 16 (62%) patients had ≥50% reduction in bleeding events on prophylaxis (overall 84% reduction). Mean 5.0 bleeding events on prophylaxis vs. 13.1 with on-demand treatment; representing a 62% reduction with prophylaxis vs. on-demand ( *p* <0.001). 16 (62%) patients had ≥50% reduction in bleeding events on prophylaxis (overall 84% reduction)
Zülfikar 2012 45	–	Multicentre registry study in Turkey	Patients with HA and inhibitors	37	On demand: median 50 U/kg aPCC every 12 h (acute bleeds), 100 U/kg aPCC every 12 h (surgical haemostasis) On demand: 85 U/kg aPCC	Median: 2 infusions	112 treatment courses; 90 for acute bleeds, 22 for surgical haemostasis. Considered success in 92% of acute bleeds and 86% surgeries
Négrier 2013 47	SURF	Open-label, non-interventional, post-authorisation study	Patients undergoing surgical intervention with aPCC	35	Perioperative: 50–100 U/kg aPCC; not exceeding single dose of 100 U/kg or daily dose of 200 U/kg. 50–100 U/kg administered every 6–12 h during or after surgery	Varied	Haemostasis 'good' or 'excellent' in 91.2% of surgical procedures; 'fair' in 8.8%
Antunes 2014 49	PROOF	Randomised comparison of on-demand vs. prophylactic aPCC	Patients with HA or HB and inhibitors receiving aPCC	36	Prophylaxis: 85 ± 15 U/kg aPCC every other day. On-demand: at discretion of investigator	12 mo	Median ABR for on-demand: 28.7; prophylaxis: 7.9 (72.5% reduction)
Ewing 2015 50	–	Retrospective chart review	Children aged ≤13 y with severe HA and inhibitors receiving aPCC prophylaxis	16	Prophylaxis: 70–100 IU/kg aPCC, 3–7 times weekly	Median (range): 9 (2.6–20.5) y	AJBR reduced from 4 (0–48) at baseline to 1 (0–7) after 1 y of prophylaxis
Négrier 2016 28	PASS	Post-authorisation safety surveillance (real-world study)	Patients with HA or HB and inhibitors receiving aPCC for 1 y	81	Prophylaxis: mean (SD) 80.5 (27.8) U/kg/d aPCC On-demand: mean (SD) 104.9 (41.9) U/kg/d aPCC	12 mo	Haemostatic effectiveness judged as ‘good/excellent’ in 90.1% patients
Windyga 2019 73	FEIBA GO	Observational study	Patients with congenital HA and inhibitors	53	Prophylaxis: median (range) 61.0 (50–98) U/kg per infusion aPCC On-demand: median (range) 56.8 (2.3–62.5) U/kg per infusion aPCC	12 mo	Mean (SD) ABR for patients with >12 mo follow-up: prophylaxis ( *n* = 21): 7.1 (9.3); on-demand ( *n* = 6): 11.4 (12.8). Mean (SD) AJBR: prophylaxis ( *n* = 21): 4.2 (5.1), on-demand ( *n* = 6): 7.3 (7.9)

Abbreviations: ABR, annualised bleeding rate; AE, adverse event; AJBR, annualised joint bleeding rate; aPCC, activated prothrombin complex concentrate; CI, confidence interval; DIC, disseminated intravascular coagulation; ECMO, extracorporeal membrane oxygenation; FEIBA, factor eight inhibitor bypass activity; HA, haemophilia A; HB, haemophilia B; ITI, immune tolerance induction; IV, intravenous; NA, not applicable; PwHI, patients with haemophilia and inhibitors; rFVIIa, recombinant activated factor VIIa; SD, standard deviation.

In line with the current approach to treatment, the Future of Immunotolerance Treatment (FIT) group recommends that all patients with inhibitors should be offered at least one attempt of ITI while under prophylaxis, using the existing management algorithm. 25 The group has proposed a hypothetical approach including non-factor therapies (NFTs), but recommends that prospective clinical studies are conducted to further explore the effect of combining these agents with FVIII in ITI. 25 As patients with inhibitors have a higher potential for bleeding-related death 23 and additional daily burden, 59 all patients should be given the opportunity to eradicate inhibitors as it is the only proven regimen to restore patient response to FVIII, which is the fundamental deficiency. aPCC is part of the original ‘Bonn Protocol’, first conceived in the 1970s in Bonn, Germany, by Dr Hans-Hermann Brackmann for bleeding prevention during ITI. 54 The original protocol includes 100 IU FVIII/kg body weight and 50 U aPCC/kg body weight twice daily until the inhibitor titre decreased to <1 BU. 54 Proposed molecular mechanisms of ITI include T-cell exhaustion/anergy, inhibition of FVIII-specific memory B cell differentiation, formation of anti-idiotypic antibodies and, more recently, the generation of FVIII-specific regulatory T cells. 60 61 rFVIIa is often regarded as the preferred substance for treatment of bleeds before and during ITI due to the potential for anamnestic response with aPCC, owing to the presence of trace amounts of FVIII that may promote a rise in inhibitor titre. 62 An anamnestic response has been reported in up to 50% of patients treated with aPCC 50 63 64 65 ; nevertheless, no compromise in aPCC prophylactic efficacy has been reported, and inhibitor titres have been shown to reduce to expected levels. 50 63 64 65 ITI registries report successful treatment in 50 to 80% of patients with HA 66 67 68 69 and in 31% of patients with HB. 67 Those patients who are not successfully tolerated or not directed to ITI are usually treated with prophylactic or on-demand doses of BPAs and/or prophylactic NFTs.

The haemostatic efficacy of aPCC may be enhanced by dose optimisation, with dosage and duration of treatment being dependent on the location and extent of bleeding, the patient's clinical condition and their response. 70 A clearly defined unit of potency is required for dosage calculations and clinical management. The potency designation of aPCC is expressed in arbitrary units: 1 unit of aPCC shortens the activated partial thromboplastin time (aPTT) of FVIII inhibitor-containing reference plasma by 50%. 58 Due to its mechanism of action, which concludes with TG, there is risk of thrombosis. To avoid this expected AE, the maximum daily dosage recommendation of aPCC for approved indications as monotherapy is 200 U/kg of body weight (100 U/kg per infusion). 27 However, there are no standardised assays for the monitoring of response to BPAs, and although global assays such as thromboelastography 71 and TG assays 72 can be useful to determine coagulation response, responses are typically evaluated by assessment of individual clinical response.

Two prospective studies assessing aPCC in the treatment of PwHI are on-going: FEIBA GO (FEIBA Global Outcome; EUPAS6691) and FEIBA STAR (FEIBA Reconstitution Volume Reduction and Faster Infusion Study; NCT02764489).

FEIBA GO is a prospective, non-interventional, multi-centre cohort study in patients with HA or HB and high-responding inhibitors treated with aPCC, with a planned 4-year observation period. The study aims to assess the real-world haemostatic effectiveness and safety in individuals with aPCC treatment in routine clinical practice. 73 Long-term prophylactic outcome data from a real-world setting will be captured in an attempt to address the lack of data in this patient group.

FEIBA STAR is a phase 3b/4, prospective, multi-centre, open-label, randomised, crossover study assessing the tolerability and safety of aPCC reconstituted in regular or 50% reduced volume and of faster infusion rates (4 and 10 U/kg/min, in comparison to the standard rate of 2 U/kg/min at the regular volume) in patients with HA or HB with inhibitors. 74 The aim of the study is to clarify if reducing infusion volumes and accelerating infusion rates for aPCC will lead to increased adherence to aPCC prophylaxis.

## Non-Factor Therapies

Despite the beneficial results of BPAs, efficacy can be inconsistent, and a subset of patients on prophylactic therapy show a poor response. 75 This has prompted the development of NFTs, with alternative mechanisms of action that aim to offer treatment options that are long-acting, subcutaneously administered, and efficacious irrespective of the presence of inhibitors.

To date, only one NFT – emicizumab (Hemlibra; Roche, Basel, Switzerland) – has been approved for routine prophylaxis to prevent or reduce the frequency of bleeding episodes in patients with congenital HA with (U.S. Food and Drug Administration [FDA] 10/2017, European Medicines Agency [EMA] 2/2018) or without FVIII inhibitors for HA of any severity (FDA 10/2018) or for severe HA only (EMA 3/2019). 76 Emicizumab is a bi-specific monoclonal immunoglobulin G antibody that bridges activated FIX and FX to replace the function of missing activated FVIII, thereby enhancing coagulation and restoring haemostasis. 77 With a half-life of approximately 30 days, emicizumab is suitable for once weekly, fortnightly or monthly prophylaxis, 78 and has demonstrated prevention of bleeds in patients with inhibitors in clinical studies. In a phase 3 trial in adult PwHI, once-weekly emicizumab prophylaxis was associated with a lower rate of treated bleeding events compared with no prophylaxis (2.9 [95% CI: 1.7–5.0] vs. 23.3 events [95% CI: 12.3–43.9]). 79 However, limited data are available for perioperative use.

Although some case studies report the successful use of emicizumab prophylaxis in conjunction with perioperative rFVIIa, 80 81 82 83 there is a lack of suitable assays for BPAs and emicizumab that allow the precise monitoring of coagulation and treatment response, necessary to guide therapy when undertaking surgical procedures. 71 72 84 85 aPTT-based clotting assays, which determine FVIII activity, are not suitable for the estimation of clotting with emicizumab when used in conjunction with BPAs, because the assay is unable to reflect the combined effect on thrombin by these products, and the sensitivity of thromboelastography is currently not sufficient for guiding therapy. However, correlations between the clinical bleeding phenotype of patients and their TG capacity have been demonstrated, and as thrombin is the final product generated by combined treatment with these products, it has been proposed that the TG assay might be a more relevant test for monitoring and guiding therapy using these regimens. A three-step protocol using TG assay has been proposed to individually tailor bypassing therapy and thereby limit AEs that may occur when combining with emicizumab. 86 87 88 89

A second consideration concerns the safety of the patients receiving emicizumab concomitantly with other haemostatic agents, like BPAs, for breakthrough bleeds. Despite high efficacy rates with emicizumab prophylaxis, 36% of patients still experienced breakthrough bleeds that may have required additional treatment. 90 However, a potential synergistic and cumulative effect between emicizumab and aPCC is thought to exist. 91 While emicizumab acts by bridging activated FIX (FIXa) and FX, allowing the coagulation cascade to continue, aPCC increases the availability of FIX/FIXa. The combination of both agents, utilising different mechanisms of action, can result in excessive TG and increased thrombosis risk. 91 In HAVEN 1, a phase 3 trial of emicizumab prophylaxis in adult PwHI, TMA was reported in three patients and TEE in two patients (cavernous sinus thrombosis and skin necrosis-superficial thrombophlebitis) who received concurrent therapy with emicizumab and aPCC for breakthrough bleeding (cumulative dose >100 U/kg/day aPCC for more than 24 hours). 92 Two of the three patients who developed TMA received both rFVIIa and aPCC (one patient received rFVIIa first then aPCC, the other patient received aPCC first then rFVIIa). The authors concluded that the TMA events were driven by the synergistic effects of high cumulative doses of aPCC in combination with emicizumab. Considering the absence of report of such AEs in this population (TMA has not previously been reported with emicizumab alone in >5,200 patients as of September 2019, including >350 patients enrolled in clinical studies as of April 2017, 93 or with aPCC alone to date 49 58 94 95 ), it has been inferred that the risk of TMA is likely to arise from novel interactions between aPCC and emicizumab 96 and most likely represents a mechanism distinct from the known processes leading to TMA. 92 Indeed, in vitro experiments with aPCC and a sequence-identical analogue of emicizumab show elevated procoagulant activity demonstrated by excessive TG with combinations of aPCC and sequence-identical analogue of emicizumab at clinically relevant doses. 91 In HAVEN 1, all TEEs occurred with concomitant aPCC and emicizumab using aPCC at doses of >100 U/kg/day for >24 hours. In contrast, no events occurred with lower-dose aPCC or for treatment durations ≤24 hours, which is now the recommended dose when used to treat patients receiving emicizumab. 93 In contrast to TMAs, TEEs have also been observed with emicizumab-treated patients who are not receiving aPCC. As of March 31, 2020, 18 cases of TEEs have been reported in patients receiving emicizumab in any setting (including in patients without inhibitors), two of which included concomitant use of aPCC exceeding 100 U/kg/day. 24 97 The use of aPCC or other haemostatic agents is unknown in the 16 additional cases. One case was reported from HAVEN 3, the phase 3 clinical trial of emicizumab for the treatment of patients with HA without inhibitors. Further clinical experience and additional research are needed to more fully elucidate the safety implications of integrating different therapies into the existing treatment landscape for PwHI.

## Other Therapies in Development

Other NFTs in development are summarised in Table 3 and include anti-tissue factor pathway inhibitors, aptamers and small interfering RNA directed against anti-thrombin III. One study points toward a benefit with concomitant use of fitusiran and aPCC, with reported dosages of aPCC of 14 to 75 U/kg (13 infusions for 6 bleeds in 3 patients; mean of 2.2 infusions per bleed) and rFVIIa of 93 to 133 µg/kg (6 infusions for 4 bleeds in 3 patients; mean of 1.5 infusions per bleed). No complications were reported in this study, 98 although a sinus vein thrombosis had occurred in a fitusiran-treated patient receiving repeated infusions of FVIII product, 12 which is suggestive of similar concerns of synergistic and cumulative effects as seen with emicizumab and concomitant aPCC. In vitro and in vivo studies suggest a therapeutic benefit with concomitant concizumab and low-dose aPCC, 99 100 and a synergistic haemostatic effect was reported with concomitant concizumab and rFVIIa in human blood under haemophilia conditions, 101 but further data are required.

**Table 3:** Therapies in development for use in PwHI

Drug name	Manufacturer	Approach	Development status a	Administration	Dose (prophylaxis)	Half-life	Laboratory parameters	Potential indications
Concizumab 126	Novo Nordisk (Bagsvaerd, Denmark)	Antibody inhibiting TFPI	Phase 3 (explorer 7) (NCT04083781; study start Oct 21, 2019)	SC	TBD (evaluated range: 0.25–0.8 mg/kg every 4 d)	T(1/2): 31.1–65.9 h (0.25–9.0 mg/kg; IV) and 74.8–116 h (1.0–3.0 mg/kg; SC) 127	Elevated D-dimer, soluble fibrin and prothrombin F _1+2_ with 0.8 mg/kg dose. Decreased fibrinogen with 0.5 and 0.8 mg/kg doses. No significant changes in platelet counts, antithrombin levels, prothrombin time, aPTT, protein C and protein S	Treatment of haemophilia A or B with or without inhibitors
Fitusiran 128	Alnylam Pharmaceuticals (Cambridge, Massachusetts, United States) and Sanofi Genzyme (Cambridge, Massachusetts, United States)	siRNA-targeting antithrombin	Phase 3 (NCT03549871, study start July 30, 2018; NCT03754790, study start Jan 9, 2019; NCT03417102, study start Feb 14, 2018)	SC	TBD	Mean elimination T(1/2): 2.6–5.3 h 129	Elevated liver enzymes	Prevent bleeding episodes in patients with haemophilia A or B
Marstacimab(PF-06741086) 107	Pfizer (New York, New York, United States)	Antibody inhibiting TFPI	Phase 3 (NCT03938792, study start Oct 2019) Phase 2 (NCT03363321, study start May 30, 2018)	SC	TBD (Evaluated range: 150–450 mg)	ND	ND	Treatment of haemophilia A and haemophilia B with and without inhibitors
BAY 1093884 130	Bayer (Leverkusen, Germany)	Antibody-inhibiting TFPI	Phase 2 (NCT03597022, study start July 24, 2018)	SC and IV	TBD	ND	ND	Severe haemophilia A or B with or without inhibitors
Eptacog alfa (AryoSeven) 104	AryoGen (Tehran, Iran)	Activated factor VIIa (biosimilar)	Phase 3 (NCT03935334, study start July 23, 2018)	IV	90–270 μg/kg	ND	ND	Haemophilia A or B with inhibitors
MC710 105	KM Biologics/Kaketsuken (Kumamoto, Japan)	Activated factor VIIa/FX	Phase 3	IV	60 or 120 μg/kg	MC710 components: FVII:C 1.8–2.6 h, FVII:Ag 3.1–3.6 h, FX:C 15.8–16.0 h, FX:Ag 22.5–26.5 h 131	Elevated D-dimer, TAT and prothrombin F _1+2_ , no change in platelet count, fibrinogen	Haemophilia A or B with inhibitors
Marzeptacog alfa (MarzAA) 106	Catalyst Biosciences (San Francisco, CA, United States)	Activated factor VIIa	Phase 2/3 (NCT03407651, study start Dec 18, 2017)	SC	TBD	3.5 h (terminal)	ND	Haemophilia A or B with inhibitors
Eptacog beta 110	HEMA Biologics/LFB-USA (Framingham, Massachusetts, United States)	Recombinant coagulation factor VIIa	Phase 3 (NCT02448680, study start Dec 2015)	IV	TBD (evaluated doses: 75 and 225 µg/kg)	1.8–2.3 h (terminal) 132	ND	Haemophilia A or B with inhibitors
SPK-8016 133	SPARK Therapeutics (Philadelphia, Pennsylvania, United States)	AAV5 viral vector gene therapy	Phase 2/3 (NCT03734588, study start Jan 30, 2019)	IV	TBD (single dose)	ND	ND	Haemophilia A with inhibitors
AMT-180 114	uniQure (Amsterdam, Netherlands)	AAV5 viral vector gene therapy with modified factor IX gene (Super9)	Preclinical	IV	TBD (single dose)	ND	ND	Haemophilia A with or without inhibitors

Abbreviations: AAV, adeno-associated virus; aPTT, activated partial thromboplastin time; IV, intravenous; ND, no data; SC, subcutaneous; TAT, thrombin–antithrombin complex; TBD, to be determined; TFPI, tissue factor pathway inhibitor.

a Status as of November 7, 2019.

Several rFVIIa compounds are in development for PwHI ( Table 3 ). An rFVIIa biosimilar protease, activated eptacog alfa (Coagil-VII; Generium), has been approved for PwHI undergoing surgery in Russia. 102 However, very little clinical data have been published to date. A series of case studies with the substance reported about the experience of 10 patients given Coagil-VII after switching from NovoSeven. There were therapeutic failures in four patients and allergic reactions in two patients, for whom it was necessary to revert to NovoSeven, and a good clinical response was achieved in those patients. 103 A second rFVIIa biosimilar is in development in PwHI (AryoSeven; Aryogen), with a similar response to rFVII (NovoSeven) reported in a clinical study. 104

Another FVIIa product, MC710, being developed by the Chemo-Sero Therapeutic Research Institute (Japan), is a 1:10 protein weight ratio mixture of plasma-derived activated FVIIa and FX. 105 In a phase 3, open-label study of 21 joint, muscle and subcutaneous bleeding episodes in 14 male patients, individuals received one or two doses of intravenously administered MC710 at 60 or 120 μg/kg once or twice (to a maximum of 180 μg/kg) for up to five bleeding episodes per patient. Nineteen episode treatments were rated ‘excellent’ or ‘effective’ 8 hours after the last treatment. 105

The rFVIIa variant marzeptacog alfa (MarzAA; Catalyst Biosciences) was designed to combine higher clot-generating activity and longer activity at the site of bleeding and therefore improve efficacy. 106 It is anticipated that the compound could be used for both subcutaneous prophylactic treatment and intravenous acute treatment, and may be valuable for patients with HB with inhibitors, or patients with HA with inhibitors who failed emicizumab. 107 108 The compound has achieved orphan status and is currently in phase 2 trials for patients with HA or HB with inhibitors; nine patients with high annual bleeding rates (ABRs) before the study (15.2–26.7 bleeds per year) have successfully completed long-term daily dosing with the compound. Seven participants experienced no bleeds with a 30-µg/kg dose and two others who escalated their dose to 60 µg/kg had clinically significant reductions in ABR and proportion of days with bleeding. No anti-drug antibodies were detected, but further safety data are needed. 109

Eptacog beta (LR769; Hema Biologics/LFB) is a transgenic recombinant human FVIIa produced in rabbits and approved by FDA on April 1, 2020 for the treatment of bleeding episodes in HA or HB patients with inhibitors. 110 In a pivotal phase 3 study of two initial dose regimens in 468 bleeding events in 27 PwHIs, both study arms met the primary endpoint of haemostatic success (evidence of cessation of bleeding). 110 The majority (85%) of the bleeding events treated with the initial 225-µg/kg dose required no further therapy. Currently, two additional phase 3 trials are investigating the use of eptacog beta in paediatric and surgical PwHIs. 111

Encouraging results from clinical trials have also stimulated considerable interest in the application of gene therapy for the treatment of haemophilia, using in vivo gene transfer to the liver using adeno-associated viral vectors. Results from recent clinical trials suggested some therapeutic expression, and in some cases a curative effect. 112 Although the presence or history of inhibitors has until recently been considered an exclusion criterion for studies with gene therapy for haemophilia, 113 gene therapy trials are now underway for haemophilia patients with inhibitors. These include SPK-8016 (Spark Therapeutics), in phase 1/2 development for patients with HA and inhibitors, 114 and AMT-180 (uniQure), in preclinical development for patients with HA with past or current inhibitors. 114

## Use of aPCC in PwHIs in the Evolving Treatment Landscape

As we enter an era of therapies for PwHIs with higher efficacy for prophylaxis, the use of BPAs is expected to decrease in such clinical settings. 115 However, the use of aPCC is still recommended in recent treatment guidelines for haemophilia. 3 4 5 6 7 Various scenarios in which the use of aPCC may remain necessary include (1) treatment of breakthrough bleeds or (2) as surgical prophylaxis in patients under NFT prophylaxis. Findings from the HAVEN 1 trial and the interim analysis of the STASEY study indicate that up to 37% of patients still experienced bleedings. 31 92 Recent data, supported by clinical experience, indicate that aPCC < 50 U/kg is sufficient to fully restore TG and has been shown to elicit a good clinical response. 24 85 116 117 118 119 (3) When following high-dose ITI regimens, such as the Bonn Protocol. For example, there are currently insufficient data to fully understand whether high-dose ITI is compatible with emicizumab; therefore, until such evidence becomes available, aPCC may represent an alternative to emicizumab as prophylaxis when on an ITI regimen. (4) For treatment of acute bleeding while following an ITI protocol with addition of emicizumab, where use of aPCC (lower dose) or rFVIIa is essential. (5) Patients with HB with inhibitors, and (6) patients with acquired HA, 25 for whom emicizumab is not indicated. 76 (7) Well-controlled patients who achieve ≥50% reduction in the number of bleeds 44 under a current aPCC prophylaxis regimen, e.g. patients with an ABR of <4 per year, or patients who have succeeded with ITI. (8) On-demand treatment in low income and developing countries, where access to new products may be limited ( Table 4 ).

**Table 4:** Scenarios for use of aPCC in the evolving treatment landscape

1	Treatment of breakthrough bleeds in patients receiving NFT prophylaxis
2	Surgical prophylaxis in patients receiving NFT
3	Prophylaxis while on high-dose ITI
4	Treatment of acute bleeds while under ITI
5	Patients with haemophilia B with inhibitors
6	Patients with acquired haemophilia A
7	Well-controlled patients achieving ≥50% reduction in bleeds with aPCC or with ABR/AJBR <4
8	On-demand treatment in low-income and developing countries with limited access to treatment

Abbreviations: ABR, annualized bleeding rate; AJBR, annualised joint bleeding rate; aPCC, activated prothrombin complex concentrate; ITI, immune tolerance induction; NFT, non-factor therapies.

## Conclusions

Since the 1970s, aPCC has been used for the treatment and prevention of acute bleeds in PwHIs and, later with rFVIIa, became standard of care. Even after more than 40 years, aPCC has a continuing role in the treatment of acute bleeds, prophylaxis, and surgical management in this patient group. Its most notable uses are for prophylaxis in patients for whom emicizumab is not available or applicable, for patients initially receiving rFVIIa who do not respond to treatment, and for low-dose treatment of breakthrough bleeds or surgery. However, there remains a need for alternative treatments for breakthrough bleeding (either spontaneous or post-trauma) and surgical applications that do not carry a risk of excess TG with concomitant usage with prophylactic treatment. As further data are generated with the newer therapeutic molecules and gene therapies for PwHI, there will be a need to identify the patient profiles that benefit most from each treatment, or combinations of treatments, for example, with the use of surrogate markers of haemostasis to enable selection of a suitable management strategy. Understanding the potential risks of unexpected AEs when integrating new therapies into the treatment armamentarium is vital, as already observed with the integration of emicizumab with aPCC. Consequently, there is a need for further exploration of potential synergistic effects between agents, of dose-ranging for the avoidance of TMA and other thrombotic risks, and for monitoring studies of combination therapies with aPCC. Considering inter-patient variability, it will be important for all haemostatic agents to remain available, with recommendations on how to use them, to ensure optimal treatment and for prevention of bleeding episodes as well as patient safety.
